# MRI with ultrahigh field strength and high-performance gradients: challenges and opportunities for clinical neuroimaging at 7 T and beyond

**DOI:** 10.1186/s41747-021-00216-2

**Published:** 2021-08-26

**Authors:** Behroze Vachha, Susie Y. Huang

**Affiliations:** 1 Department of Radiology, Memorial Sloan Kettering Cancer Center, 1275 York Ave, New York, NY 10065 USA; 2 Athinoula A. Martinos Center for Biomedical Imaging, Department of Radiology, Massachusetts General Hospital, 149 13th Street, Room 2301, Charlestown, MA 02129 USA

**Keywords:** Epilepsy, Magnetic fields, Magnetic resonance imaging, Multiple sclerosis, Neuroimaging

## Abstract

Research in ultrahigh magnetic field strength combined with ultrahigh and ultrafast gradient technology has provided enormous gains in sensitivity, resolution, and contrast for neuroimaging. This article provides an overview of the technical advantages and challenges of performing clinical neuroimaging studies at ultrahigh magnetic field strength combined with ultrahigh and ultrafast gradient technology. Emerging clinical applications of 7-T MRI and state-of-the-art gradient systems equipped with up to 300 mT/m gradient strength are reviewed, and the impact and benefits of such advances to anatomical, structural and functional MRI are discussed in a variety of neurological conditions. Finally, an outlook and future directions for ultrahigh field MRI combined with ultrahigh and ultrafast gradient technology in neuroimaging are examined.

## Key points

- Increased signal-to-noise ratio and high spatial resolution conferred by ultrahigh field magnetic resonance imaging (MRI) can be used to improve the delineation of small anatomical structures and subtle pathology.
- The challenges associated with ultrahigh field MRI include artifacts associated with increased susceptibility effect, inhomogeneity of the radiofrequency transmit field, and increased radiofrequency energy deposition in tissues.
- High-performance gradient systems equipped with high maximum gradient amplitudes and slew rates enable increased spatial resolution and faster acquisitions.
- Technical limitations of high-performance gradient systems include the biological effects of rapidly switching, large magnetic fields on the human body, which can induce peripheral nerve stimulation.

## Background

The last 20 years have seen a push in the development of magnetic resonance imaging (MRI) scanner technology with remarkable advances in field strength and progress in gradient coil design and performance. Clinical MRI scanners with magnetic field strengths up to 7 T and gradient strengths up to 80 mT/m with a peak slew rate of 200 T/m/s have now become the state-of-the-art imaging technology for clinical and research studies of the brain. For neuroimaging, research in ultrahigh magnetic field strength and ultrahigh, ultra-fast gradient technology has led to a multitude of benefits for high-resolution anatomic, structural, and functional imaging, opening an array of opportunities for changing the way that patients are imaged and evaluated, and providing new insights into the pathophysiology of neurological diseases.

## Ultrahigh field MRI

### Technological overview and state-of-the-art

Since the first ultrahigh field human MRI images were obtained at 8 T in 1998 [1], ultrahigh field MRI has gained increasing traction worldwide through the development and dissemination of 7-T scanners for human imaging. The promise of ultrahigh field MRI in pushing the limits of sensitivity and spatial resolution, initially demonstrated in the research arena, has been realized through the commercialization of 7-T MRI scanners by the major MRI scanner vendors, culminating in Food and Drug Administration-approved scanners for clinical use in 2017 through the MAGNETOM Terra 7-T MRI scanner (Siemens Healthineers, Erlangen, Germany) and in 2020 through the Signa 7-T MRI scanner (General Electric Healthcare, Chicago, IL, USA). Based on the success of current 7-T and 9.4-T human MRI scanners, concerted efforts are now underway to develop the next generation of ultrahigh field MRI scanners for human use at 10.5-T and 11.7-T, with field strengths up to 20 T deemed feasible, opening up new opportunities for unprecedented sensitivity and specificity for human imaging [2].

### Challenges and opportunities of imaging at higher field

The benefits and challenges of imaging at higher static magnetic field strength directly follow from the dependence on magnetic field strength of the physical properties of tissues and other magnetic fields involved in image formation. The main advantage of increasing the magnetic field strength for MRI is increased sensitivity. The signal scales greater than linearly with magnetic field strength [3]. The resulting increase in signal-to-noise ratio (SNR) can be used to increase spatial resolution and improve the delineation of small anatomical structures and subtle pathology. For example, the spatial resolution of anatomical T1-weighted images can be pushed to the sub-millimeter level, enabling better estimation of cortical thickness through the reduction of partial volume effects [4, 5] and improving the segmentation of the hippocampal subfields [6, 7] and amygdalar nuclei [8, 9].

**Fig. 1:**
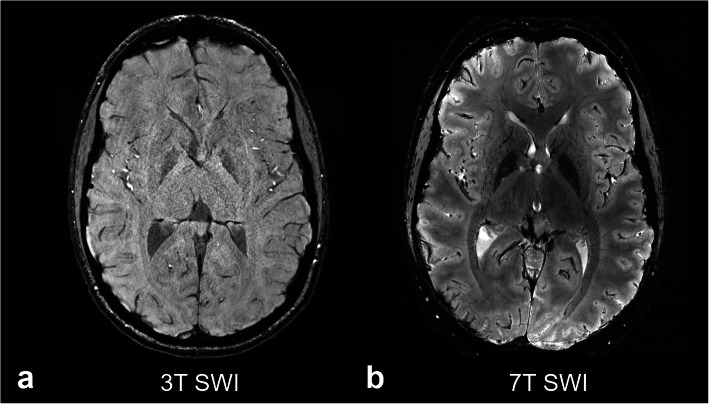
Comparison of susceptibility-weighted imaging (SWI) using 3-T and 7-T magnetic resonance imaging. Axial SWI images are shown at the level of the basal ganglia in a healthy individual acquired at 3 T (**a**) and 7 T (**b**). At higher field strength, the shorter T2* relaxation times result in the greater SWI sensitivity to magnetic susceptibility differences in the blood vessels and mineralization in the basal ganglia, particularly in the globus pallidus

Tissue relaxation properties also change with magnetic field strength, with an expected prolongation of the longitudinal relaxation time T1 and shortening of the transverse relaxation times T2 and T2* at higher field. These changes in relaxation properties along with increased SNR benefit certain applications; for example, longer T1 relaxation times offer better signal-to-noise ratio for applications such time-of-flight (TOF) magnetic resonance (MR) angiography [10] and arterial spin labeling [11], whereas shorter T2* relaxation times provide greater image contrast for susceptibility-weighted imaging (SWI) (Fig. 1) and for detecting functional activation by blood oxygenation-level dependent (BOLD) functional MRI (fMRI) [12]. Indeed, a major advantage of imaging at higher field is the improvement in time-series SNR and greater sensitivity to temporal correlations in the BOLD signal, which can capture previously unrecognized nodes in functional networks [13].

The challenges associated with ultrahigh field MRI include increased susceptibility-induced distortions at higher field, inhomogeneity of the radiofrequency (RF) transmit field, and increased RF energy deposition in tissues. As field strength increases, the resonant frequency increases and is approximately 300 MHz at 7 T. Accounting for the dielectric properties of tissue, the wavelength of the RF transmission field at 7 T is approximately 11 cm, which is shorter than the dimensions of the human body, leading to noticeable variations in the RF transmission field due to the creation of standing waves. Beyond increased transmit inhomogeneity, the amount of RF power deposited in tissue increases with field strength, resulting in higher specific absorption rate (SAR). In practice, clinical 7-T MRI uses only local RF transmit coils, for which the International Electrotechnical Commission has advised a higher threshold of 10 W/kg for local SAR compared to the whole-body SAR limit of 2 W/kg [14]. The use of MR sequences with high power deposition (*e.g.*, fast spin-echo and inversion-recovery) is limited at higher field strengths. Spatial variations of the RF transmit field due to standing waves can also produce localized heating. Metallic implants may also lead to heating due to RF absorption, although many studies have shown that heating may be less of a problem than initially anticipated, with only minor temperature changes incurred by small, passive implants (*e.g.*, hemostatic clips, aneurysm clips, prosthetic valves, vascular access ports, and ossicular and ocular implants) [15–17].

At higher field strengths, the spectral separation between different chemical species increases, leading to higher spectral resolution, which is beneficial for MR spectroscopy but can lead to greater imaging artifacts due to the increased chemical shift and susceptibility effect. Susceptibility artifacts can adversely affect image quality in ultrahigh field MRI, especially in gradient-echo imaging. Susceptibility-induced artifacts occur as a result of microscopic gradients or variations in the magnetic field strength near interfaces of different magnetic susceptibility and result in signal loss and distortion at air-bone and air-tissue interfaces, which can be particularly pronounced at 7 T and higher fields. On the other hand, the increased susceptibility effect at higher field may be used for improved diagnostic efficacy, such as increased sensitivity to microhemorrhages and better delineation of the substantia nigra. Finally, while the time-series SNR increases at higher field, the relative contribution of physiological noise from factors such as cardiac pulsation and respiratory motion also increases at 7 T compared to lower field strengths [13], necessitating strategies for reducing the effects of physiological noise [18], which are particularly important for fMRI studies at higher field strengths [19].

### Clinical applications of ultrahigh field imaging

The high spatial resolution achievable using ultrahigh field imaging has been used to study various conditions including epilepsy, multiple sclerosis, brain tumors, cerebrovascular diseases, and degenerative disorders. The following sections discuss the current state of research and potential future direction within these clinical fields.

#### Epilepsy

**Fig. 2:**
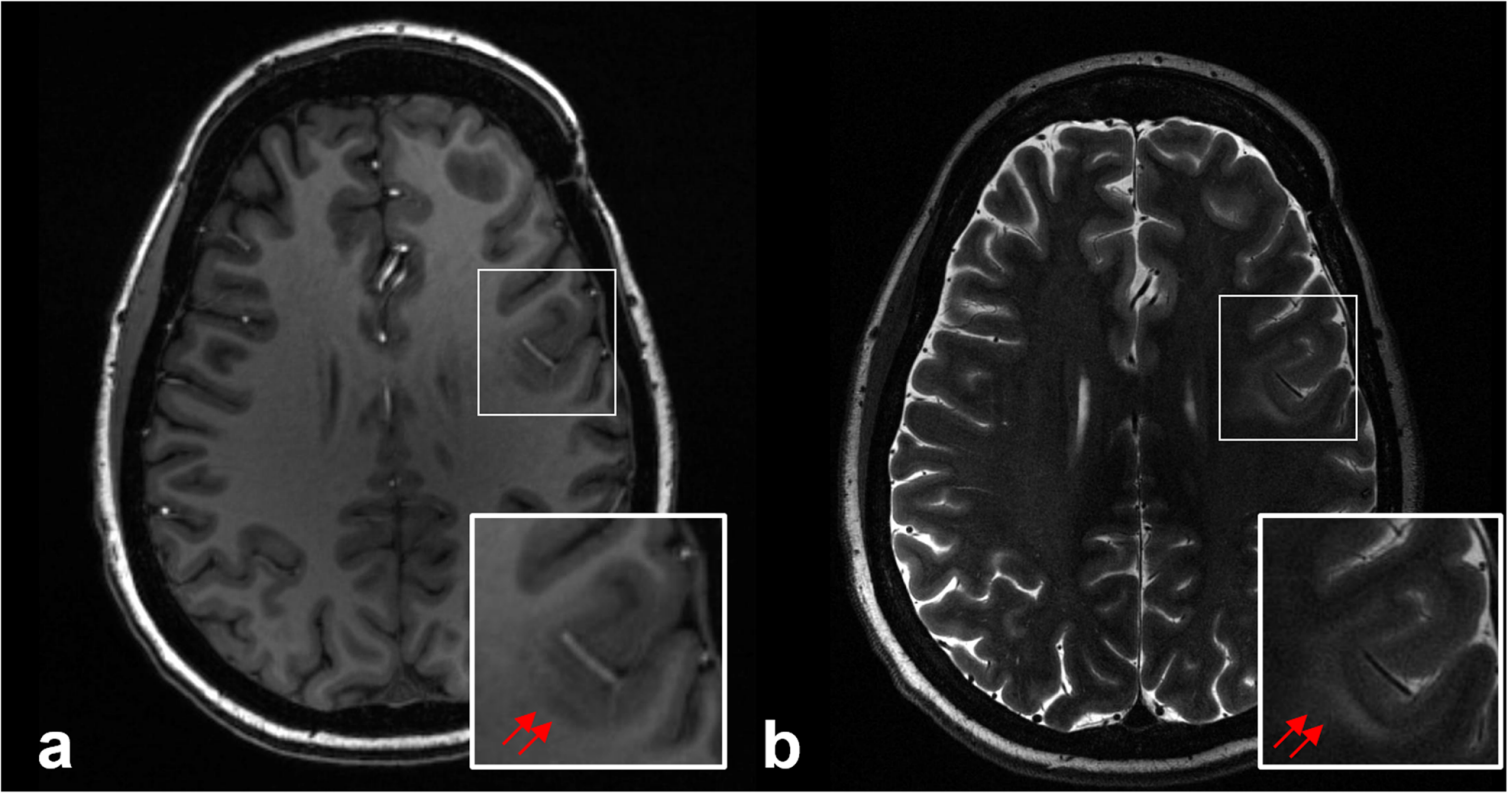
Anatomical delineation of focal cortical dysplasia at 7 T. Axial T1-weighted (**a**) and T2-weighted (**b**) images acquired at 7 T demonstrate blurring of the gray-white matter junction (double arrows), abnormally thick malformed cortex, and an indistinct cortical ribbon in the left inferior frontal gyrus, consistent with focal cortical dysplasia. There is T2 hyperintensity in the subjacent white matter reflecting the white matter component of the dysplasia

**Fig. 3:**
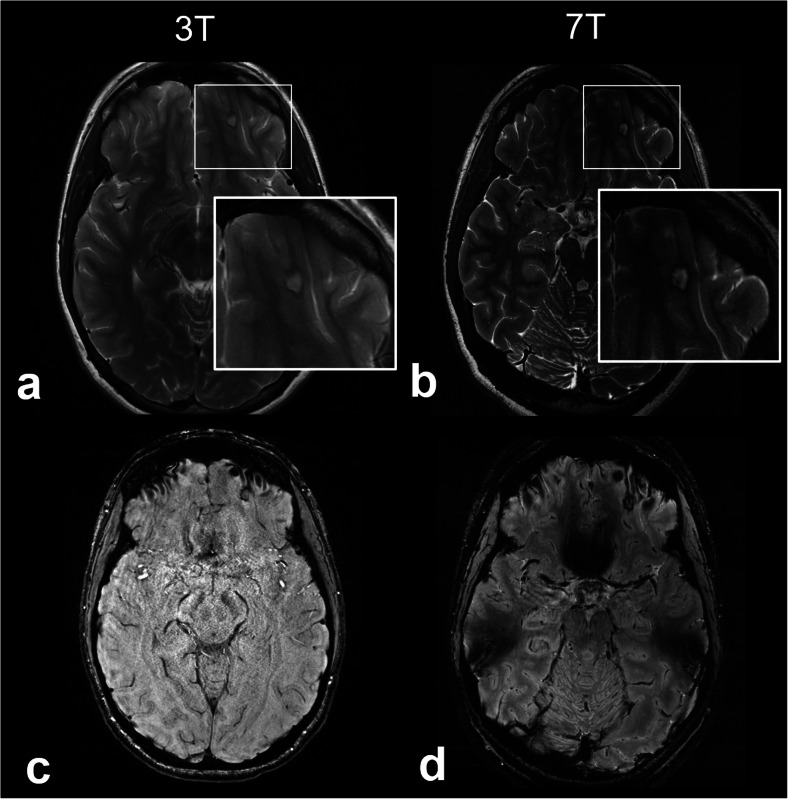
Cavernous malformation on 3-T and 7-T magnetic resonance imaging. Axial T2-weighted images acquired at 3 T (**a**) and 7 T (**b**) show a 6-mm cortical lesion in the left orbitofrontal gyrus with central T2 hyperintensity and peripheral T2 hypointensity. Axial susceptibility-weighted images acquired at 3 T (**c**) and 7 T (**d**) show marked susceptibility effect within the rim of the lesion. The 7-T images have higher signal-to-noise ratio and better delineate the lesion compared to the 3-T images. The lesion was resected and found to be a cavernous malformation on histopathology

**Fig. 4:**
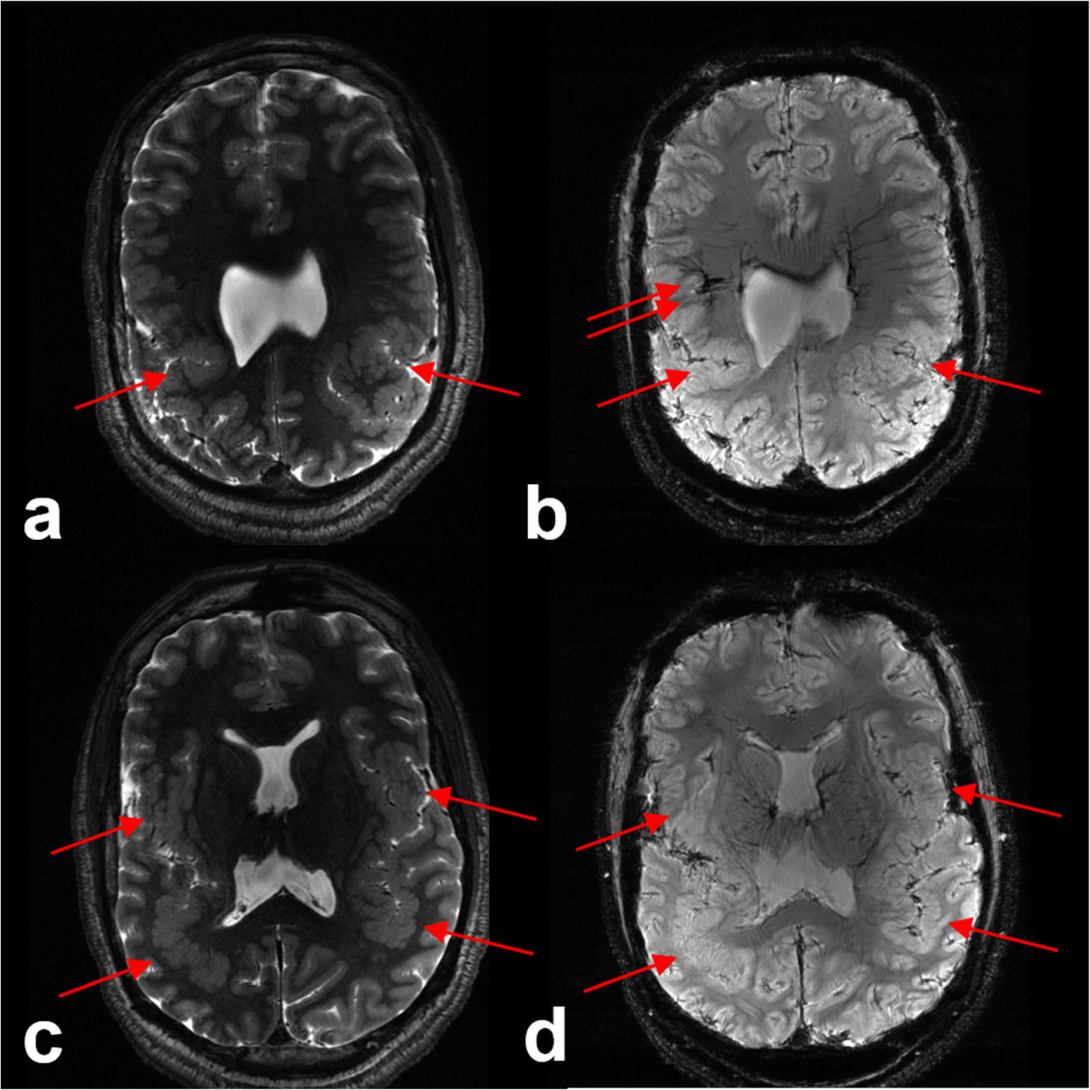
Sensitivity of 7T magnetic resonance imaging for polymicrogyria. Axial T2-weighted and susceptibility-weighted images at the level of the centrum semiovale (**a**, **b**) and lateral ventricles (**c**, **d**) demonstrate simplification of the gyral folding pattern involving the perisylvian cortex bilaterally, including the posterior temporal lobes and parietal lobes (red arrows), with thickening of the cortex and nodularity, consistent with polymicrogyria. There is also an abnormally large draining vessel on the right (**b**, red arrow), which may represent a developmental venous anomaly

In patients with medically refractory epilepsy, surgery may offer the best chances for seizure freedom [20]. Neuroimaging plays a key role in identifying the epileptogenic focus in the presurgical epilepsy work-up. However, approximately one-third of patients with focal epilepsy do not have an identifiable lesion on 1.5-T or 3-T MRI [21]. Improved SNR and the exquisitely high spatial resolution conferred by ultrahigh field imaging may help in localizing cryptogenic seizure-onset zones in patients with epilepsy that may have been missed at conventional field strengths [22, 23] (Fig. 2). Feldman et al. demonstrated that 7-T MRI revealed abnormalities of epileptogenic potential in 25 of 37 patients with focal epilepsy who had non-lesional diagnostic 1.5-T or 3-T MRI scans; 15 of these abnormalities corresponded to the clinically suspected seizure-onset site, and the detection of some abnormalities altered subsequent management [24]. SWI sequences at 7 T in this study were highly sensitive for the detection of developmental venous anomalies, cavernous malformations (Fig. 3), and polymicrogyria (Fig. 4), which either colocalized to electroencephalography data or were associated with suspected site of seizure onset [24]. These findings were supported by a prior study that found a greater number of cavernous malformations using T2*-weighted gradient-echo imaging at 7 T compared to 1.5 T [25].

In a large cohort of 67 patients with pharmacoresistant epilepsy and nonlesional 3-T MRI undergoing presurgical evaluation, Wang et al. reported that an unaided visual review of 7-T images detected previously unappreciated subtle lesions in 22% (15/67) of patients with the total yield increasing to 43% when aided by post processing of the 7-T T1-weighted magnetization-prepared two rapid acquisition gradient-echo sequence using the morphometric analysis program (MAP); the majority of lesions detected were focal cortical dysplasia [26]. The authors reported that 7-T MAP yielded 25% more lesions than 3-T MAP and demonstrated increased conspicuity in 46% [26].

Asymmetric distribution of perivascular spaces was reported in epilepsy patients compared to healthy controls when quantified on 7-T MRI scans. In the majority of the epilepsy patients, the asymmetry was greater in the same lobe as the suspected seizure onset zone. The authors suggested that the quantification of perivascular spaces at 7 T could provide an important imaging biomarker to study the effects of epilepsy on the brain and assist in localizing the seizure onset zone in surgical planning [27].

Several studies have demonstrated the excellent delineation of hippocampal morphology and internal structures at 7 T. For example, Feldman et al. found improved detection of hippocampal abnormalities in 18 patients at 7 T, and the abnormality was concordant with electroencephalography and clinical findings in 12 of the 18 patients [24]. Similarly, another study was able to demonstrate pathology in cortical area (CA) subfields using ultrahigh field MRI in a small cohort of patients with temporal lobe epilepsy that correlated with histopathological findings [28]. An evaluation of hippocampal internal structure revealed asymmetry in hippocampal vessel density both in neocortical and mesial temporal lobe epilepsy compared to healthy controls, with asymmetrically decreased vessel wall density ipsilateral to the suspected seizure onset zone [29]. Volumetric analyses and magnetic resonance spectroscopy at 7 T identified hippocampal subfield atrophy commonly affecting CA3 and altered metabolite concentrations (mainly reduced glutamine levels) in the hippocampus of patients with temporal lobe epilepsy [30]. Although the volume and metabolite deviations did not consistently lateralize the epileptogenic hippocampus, lower subiculum volumes and glutamine concentrations correlated with impaired verbal memory performance [30]. Using resting state fMRI at 7 T, Shah et al. [31] demonstrated functional network asymmetry within the mesial temporal lobe that was able to distinguish between temporal lobe epilepsy subtypes, suggesting that the improved SNR and resolution at 7 T has the potential to improve localization of underlying brain network disruptions in temporal lobe epilepsy patients who are candidates for surgical resection.

In summary, the higher SNR and increased spatial resolution afforded by 7-T MRI has been shown to improve the sensitivity of detecting epileptogenic lesions and may aid in the identification of morphological variations associated with suspected seizure onset zones.

#### Multiple sclerosis

The increased sensitivity of lesion detection and better characterization of lesion pathology at 7 T promises to provide better differentiation of multiple sclerosis (MS) from other disorders [32]. The identification of a central vein inside white matter lesions on MRI, the central vein sign (CVS), has been proposed as an imaging biomarker in the diagnosis of MS, with many studies suggesting that the presence of the CVS accurately differentiates MS from its mimics [33, 34]. In a study of 7 patients with MS and a total of 358 white matter lesions, Tallantyre et al. [35] showed that the CVS was identified in 87% of lesions at 7 T compared to 45% of visible lesions at 3 T using T2*-weighted sequences. A subsequent study by the same group demonstrated that at 7 T, 80% of lesions in MS patients were perivenous compared to 19% of lesions in non-MS patients, and that 7-T T2*-weighted MRI reliably distinguished patients with clinically definite MS from those without clinical MS based on the percentage of lesions with the CVS [36]. A study using SWI and magnetization-prepared fluid attenuated inversion-recovery (FLAIR) images at 7 T demonstrated that at a threshold of 67%, perivenous nonconfluent white matter lesions of 3 mm in length were able to differentiate relapsing remitting MS patients from healthy controls with a sensitivity of 94% and a specificity of 100% [37]. Despite these findings, recent meta-analyses have suggested that while the higher SNR and contrast-to-noise ratio of 7-T scanners result in slightly better delineation and detection of the CVS (pooled proportion of central veins detected at 7 T = 0.82), scanning at 3 T might be sufficient in this regard (pooled proportion of central veins detected = 0.74), while the use of 1.5-T scanners showed a statistically significant reduction in CVS detection (pooled proportion of central veins detected = 0.58) [38].

**Fig. 5:**
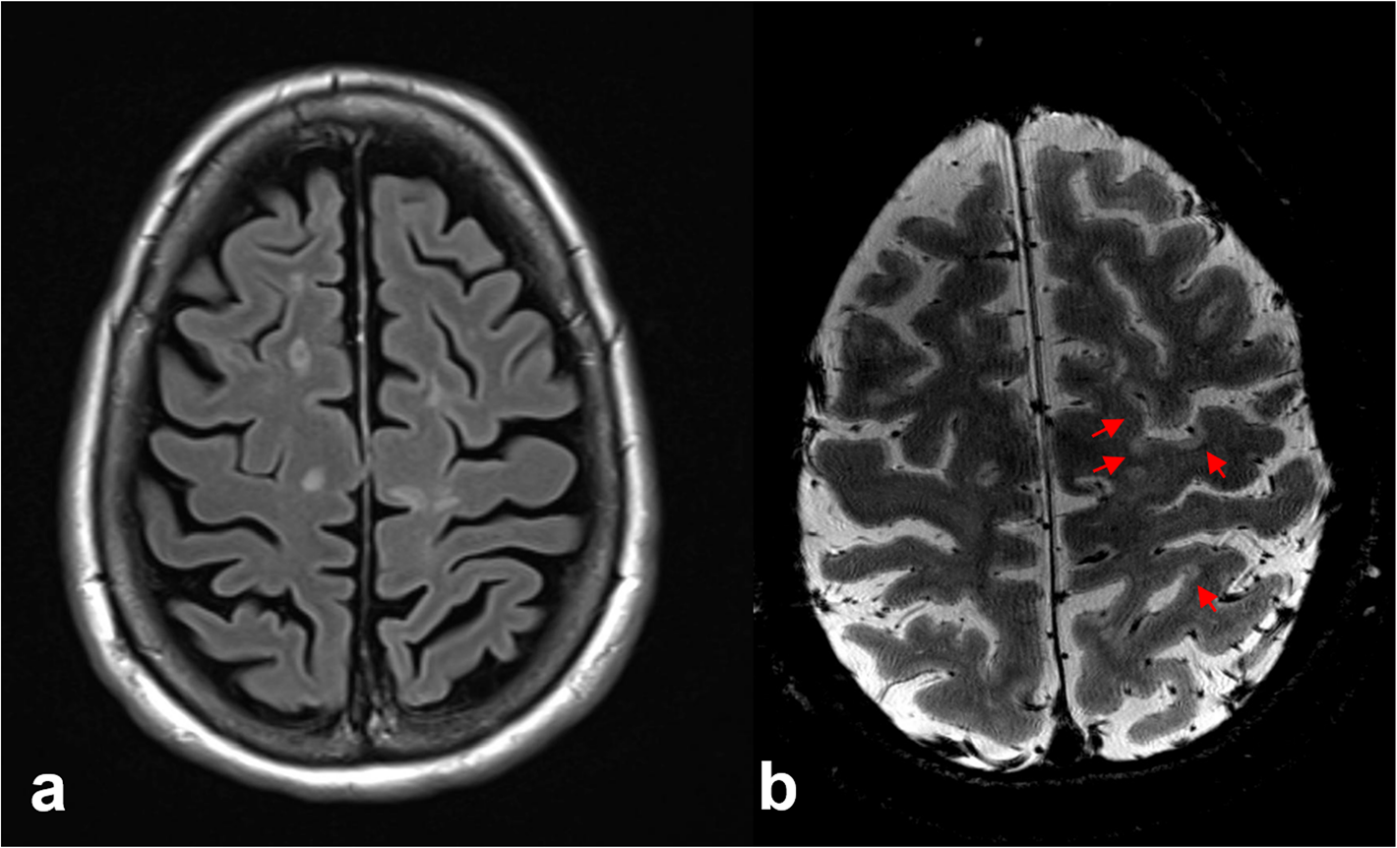
Cortical lesions in multiple sclerosis on 3-T and 7-T magnetic resonance imaging. Axial fluid-attenuated inversion-recovery (FLAIR) image acquired at 3 T (**a**) and axial T2*-weighted image acquired at 7 T (**b**). The 7-T image shows many more cortical lesions (red arrows), which are not visible on the axial FLAIR (**a**) or T2-weighted image (not shown) at 3 T, due to a combination of increased contrast and spatial resolution at 7 T

Beyond the detection of the CVS, the increased spatial resolution at 7 T has enabled the detection of cortical gray matter lesions in MS, which are frequently observed on histopathological examination but are difficult to detect at lower field strengths due to diminished contrast and lower spatial resolution (Fig. 5) [39–42]. Cocozza et al. [40] demonstrated that cortical lesions can be identified at 7 T in MS using a three-dimensional (3D)-T1-weighted volume that was acquired in a clinically feasible time and that was comparable in performance to that achieved using a T2*-weighted sequence. In a longitudinal study using a quantitative approach that combined T2*-weighted gradient-echo acquisitions at 7 T with a surface-based analysis, Treaba et al. [42] demonstrated that cortical lesions in MS preferentially developed intracortically and within the cerebral sulci, and that their accumulation was overall independent of white matter lesion accrual, suggesting that the pathogenesis of these lesions are driven by inflammatory cerebrospinal fluid (CSF)-related processes. The authors also reported that cortical lesion accumulation independently predicted neurologic disability progression [42].

The contribution of 7-T MRI for detecting MS white matter lesions is less robust than that for the cortex [43] with ongoing research in the optimization of sequences to reduce limitations secondary to high SAR and to reduce RF field inhomogeneities [43–45]. T2*, FLAIR, magnetization transfer imaging, and magnetization-prepared rapid gradient-echo have been suggested as currently recommended sequences to visualize white matter lesions at 7 T [43, 46, 47].

The increased spatial resolution and high SNR afforded by 7-T MRI could also be beneficial in imaging demyelinating lesions of the spinal cord, which remains a challenge at lower field strengths. For example, a small study that compared spinal cord imaging of MS patients at 7 T *versus* 3 T demonstrated better visualization of spinal cord anatomy and 50% improvement in detection of MS spinal cord lesions at 7 T compared to 3 T [48]. Ouellette et al. [49] used 7-T MRI to characterize gray and white matter pathology in the cervical spinal cord of patients with early relapsing-remitting and secondary progressive MS and reported that spinal cord lesions were localized nearest to the subpial surfaces for those with relapsing-remitting MS, whereas lesions in progressive disease were located near the CSF surface of the central canal. This observation lends support to CSF-mediated pathogenic mechanisms in lesion development that may differ between MS subtypes. Despite the potential theoretical benefits of imaging the spinal cord in MS at ultrahigh field, technical challenges remain, including the lack of dedicated RF coils that may compromise image quality; small variations in tissue susceptibility that are enhanced at 7 T; and motion artifacts due to respiration, cardiac movement, swallowing and CSF flow [32, 43].

In summary, even though the use of ultrahigh field MRI is not yet part of standard-of-care imaging protocols in MS, it has allowed for improved imaging characterization and identification of the different aspects of MS pathophysiology, thereby offering new insights into inter-individual variability and disease evolution.

#### Brain tumors

**Fig. 6:**
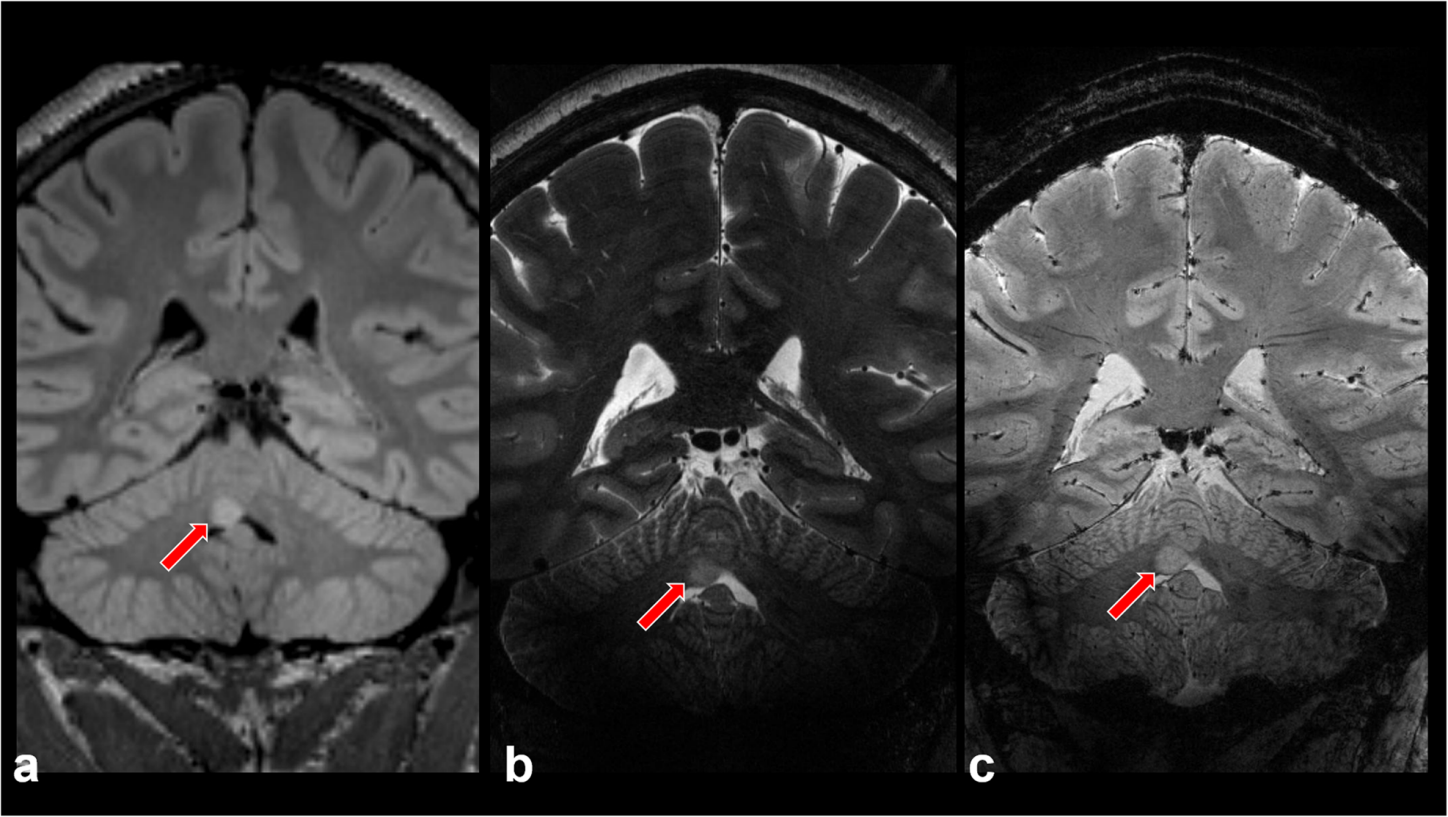
Hamartoma identified on 3-T magnetic resonance imaging and further characterized at 7 T. Coronal T2-weighted fluid-attenuated inversion-recovery image at 3 T (**a**) demonstrates a hyperintense mass (red arrows) in the right cerebellar vermis. Coronal T2-weighted (**b**) and coronal susceptibility-weighted image (**c**) obtained at 7T show no evidence of microvascularity within the lesion

Several studies suggest that 7-T MRI can provide superior information regarding microvascularity and necrosis than 1.5-T and 3-T MRI and could facilitate early stratification of patients with gliomas (Fig. 6), in accordance with recent World Health Organization grading schemes [50–53]. For example, Moenninghoff et al. [51] demonstrated higher tumor microvascularity in high-grade gliomas compared with low-grade lesions using 7-T SWI. These results concurred with those of Grabner et al. [54] who found that a local image variance technique for quantification of hypointense microvascular SWI structures at 7 T was able to differentiate diffuse infiltrative gliomas based on tumor grade and isocitrate dehydrogenase (IDH) mutational status. Christofordis et al. [55] demonstrated that tumoral pseudoblush obtained using gradient-echo at ultrahigh field correlated histologically with increased microvascularity and overall tumor grade.

TOF MR angiography at 7 T has recently been used to characterize intratumoral vessels successfully in a small sample of 12 patients with gliomas [56]. The advantages of TOF angiography at 7 T compared to lower field strengths (increased SNR, longer T1 relaxation times augmenting vessel-tissue contrast and hyperintense arterial vasculature) suggest it may be a useful noninvasive method of grading gliomas since higher grade gliomas tend to display increased angiogenesis. Furthermore, 7-T vascular imaging has been leveraged to study longitudinal glioma microvasculature changes after antiangiogenic therapy, with a potential role in the noninvasive monitoring of antiangiogenic therapy response in patients with aggressive brain tumors [57].

High-resolution FLAIR images at 7 T were compared with clinical FLAIR sequences at 3 T in patients with glioblastoma prior to radiotherapy planning and yielded significantly higher SNR in white matter and better contrast between gray and white matter compared to clinical FLAIR sequences [58]. The authors suggested that the better visibility of anatomical borders afforded by improved gray matter and white matter contrast at 7-T FLAIR could be leveraged to improve the delineation of brain structures at risk during treatment planning. A recent study demonstrated that sodium (^23^Na) MRI at 7 T correlated with isocitrate dehydrogenase (IDH) mutation status in glioma patients with potential application in image-guided biopsy, surgery, and radiotherapy [59]. The authors also reported a successive decrease of ^23^Na concentration from regions of central necrosis to normal appearing white matter suggesting a correlation with tumor infiltration.

Magnetic resonance spectroscopy (MRS) at higher field strengths improves SNR and spectral dispersion, leading to improved sensitivity and specificity, respectively, to different chemical species and metabolites. MRS has the potential to improve the detection of metabolites for tumor characterization; however, the technical challenges of increased SAR, magnetic field inhomogeneities, and fast T2*-relaxation can impose constraints on the acquisition of MRS data at 7 T [60–62]. Despite these limitations, earlier research at 7 T was able to demonstrate the application of a short-echo spin-echo MRS sequence to detect characteristic differences in regions of tumor *versus* normal appearing brain and successfully discriminate between short-echo metabolites, particularly glutamate and glutamine, although with low spatial resolutio n[63]; this differentiation remains elusive at field strengths of 3 T and below [60]. The last decade has seen different MRS acquisition approaches to offset some of the challenges at ultrahigh field imaging [60, 64–67]. For example, Hangel et al. [66] used 7-T MRS combined with patch-based super-resolution (PBSR) reconstruction in ten glioma patients that resulted in the identification of complex metabolic activities, which were in topographic agreement with tracer uptake on PET. In a subsequent study, Hangel et al. [60] successfully demonstrated metabolic differences between tumor regions and peritumoral tissues for multiple metabolites using a fast high-resolution whole-brain 3D MRS method in 23 patients with histologically verified high grade gliomas. Although 2-hydroxyglutarate (2HG) was not satisfactorily quantified in this study, other studies have demonstrated detection of 2HG at ultrahigh field [64, 68, 69]. For example, using a dual-read out alternated gradients echo-planar spectroscopic imaging (DRAG-EPSI) approach at 7 T, An et. al. demonstrated reliable high-resolution 2HG imaging in a small cohort of glioma patients [64]. Bisdas et al. demonstrated 2HG using MRS at 9.4 T [69]. However, additional validation studies using larger sample sizes are needed for quantification of 2HG and other cancer metabolites at ultrahigh field.

Chemical exchange saturation transfer imaging (CEST), a novel contrast technique that can detect low concentration metabolites with higher sensitivity than MRS, has been used both at clinical and ultrahigh field strengths to differentiate gliomas [70–74]. Paech et al. [73] demonstrated that relaxation-compensated amide proton transfer (APT) MRI was significantly correlated with patient overall survival and progression-free survival in newly diagnosed high-grade glioma patients. CEST-derived contrasts at 7 T, particularly Nuclear Overhauser effect (NOE) imaging as well as downfield-NOE-suppressed APT, were demonstrated to be significant predictors of early progression after first-line therapy in twenty previously untreated glioblastoma patients [75]. Meissner et al. demonstrated that relaxation-compensated relayed NOE (rNOE)-mediated CEST imaging at 7 T was able to discriminate responders from non-responders immediately after the end of chemoradiotherapy in 12 patients with gliomas which was at least 4 weeks earlier than the standard clinical evaluation by Response Assessment in Neuro-Oncology, suggesting that CEST MRI has the potential to enable early response assessment in glioma patients [72]. Paech et al. [74] demonstrated that relaxation-compensated multipool CEST MRI, particularly downfield-rNOE-suppressed-APT imaging, enabled the prediction of IDH mutation status and differentiation of high-grade from low-grade gliomas, suggesting the potential to use this technique as a noninvasive imaging biomarker in the diagnostic workup of these tumors. A recent study showed that CEST signal intensities at 7 T are dependent on the anatomic location of high-grade gliomas such that NOE showed significant dependence on subventricular zone contact while amide CEST signals depended on the hemispheric location of the glioma [70]. These recent findings, along with associated contaminants and the occasional difficulty in how to interpret these signals, suggest the need for additional investigation before the adoption of these techniques into routine clinical practice [53].

In summary, ultrahigh field MRI improves the detection of small metastases, delineation of tumor extent, characterization of the intratumoral microenvironment, and detection of post-treatment effects when compared to conventional strength MRI. However, technical challenges related to MRS and novel CEST techniques remain and warrant additional research.

#### Neurodegenerative disorders

Studies at 7 T within the field of neurodegenerative disorders have focused mainly on (1) dementia including Alzheimer’s disease (AD) and vascular dementia and (2) Parkinson’s disease (PD). Although hyperphosphorylated tau and amyloid beta are considered key mediators in the pathogenesis of AD, an increasing number of studies suggest that altered iron metabolism also plays an important role [76–80]. T2*-weighted MRI at 7 T performed on postmortem frontal cortex of patients with early-onset and late-onset AD as well as controls demonstrated severe disruption of the cortical lamination in AD patients, which correlated with changes in cortical myelin and iron accumulation [76]. A subsequent study by the same group evaluated postmortem medial temporal lobe in AD and controls and demonstrated more variety in visible cortical lamination in AD patients as well as severely distorted cortical lamination in advanced stage AD patients; the changes in appearance of visible cortical lamination were associated with diffuse cortical iron alterations and iron deposition [78].

A series of studies have used 7-T MRI to delineate the pattern of atrophy of different substructures of the hippocampus in AD with implications for understanding the neuropathogenesis involved in memory loss in these patients [81–84]. Bouvy et al. [85] demonstrated a topographical association between a high degree of juxtacortical perivascular space dilation adjacent to cortical cerebral microbleeds in amnestic mild cognitive impairment and early AD patients that may be due to impaired drainage of interstitial fluid related to perivascular amyloid deposition. The authors suggested that dilated perivascular spaces at ultrahigh field may act as an indirect neuroimaging marker for cerebral amyloid angiopathy in AD.

Deep brain stimulation (DBS) of target nuclei such as the subthalamic nucleus, globus pallidus pars interna, and ventralis intermedius nucleus of the thalamus is a widely performed surgical treatment for patients with PD in which an electrode is placed to stimulate the motor components while avoiding the associative and limbic components [86]. Improved imaging contrast, resolution, and SNR at 7 T afford improved visualization of DBS target nuclei compared to standard 1.5-T or 3-T scanners [87–91]. Patriat et al. [89] used tractography-based parcellation at 7 T in PD patients prior to DBS surgery to segment the globus pallidus pars interna into motor, associative, and limbic components and identified functional territories that were organized in a reproducible manner. Similarly, using a structural connectivity-based parcellation protocol, the subthalamic nucleus’ connections to the motor, limbic, and associative cortical areas were used to map the individual subcomponents of the nucleus in patients with idiopathic PD [90], further reinforcing the role of ultrahigh field imaging in facilitating individualized planning of deep brain stimulation surgery.

Differences in anatomical patterns of the substantia nigra between PD patients and healthy volunteers using 7-T MRI have also been described [92], which could allow for an earlier diagnosis of the disease. A recent study demonstrated significant changes in subthalamic nucleus morphology (lower volume) and microstructural organization (lower fractional anisotropy) in patients with mild-moderate PD compared with healthy older adults, suggesting that neurodegenerative changes in PD extend beyond the substantia nigra pars compacta to involve the hyperdirect and indirect basal ganglia pathways [93]. Poston et al. leveraged quantitative susceptibility mapping at 7 T to segment individual midbrain nuclei that are associated with PD progression in 32 participants with PD [94]. The authors reported that smaller substantia nigra volumes in mild-moderate PD correlated with longer disease duration and more severe bradykinesia-rigidity scores, but not to tremor or postural instability scores. Finally, ultrahigh field imaging has been used to identify changes in hippocampal subfields in patients with PD, adding to our understanding of the underlying neuroanatomical mechanisms of episodic memory impairments seen in PD [95].

In summary, increased image resolution at 7 T MRI enables the visualization of small structures of interest in neurodegenerative pathophysiology including cortical layers, hippocampal subfields, brainstem nuclei, plaques, and microbleeds. 7-T MRI has been successfully employed to detect biomarkers of disease in both AD and PD and may offer a powerful complement to other noninvasive imaging modalities in the evaluation of dementia.

#### Cerebrovascular diseases

MRI remains the most sensitive imaging technique to detect acute infarction. Clinically feasible stroke imaging protocols have been designed to image subacute and chronic infarcts at 7 T [96]. The exclusion of diffusion-weighted imaging (DWI) as part of the protocol precludes its use in current clinical practice for the evaluation of acute stroke patients [97]. Despite this, the high SNR, high spatial resolution, and high CNR afforded by ultrahigh field MRI can be used to evaluate small cerebrovascular lesions (such as cortical microinfarcts) and vasculature on the submillimeter scale, thereby allowing better characterization of cerebrovascular disease including stroke using 7 T compared to lower field strengths [52, 97, 98].

MR angiography at 7 T has been used successfully to image occlusive changes in small vessels such as the lenticulostriate arteries in acute and chronic strokes; these vessels are difficult to image with conventional modalities [99, 100]. Further, when images of patients with subacute and chronic strokes were compared at 7 T and 3 T, the higher spatial resolution of 7-T scanners revealed subtle features of infarct morphology compared to the lower field MR images [96].

Intracranial atherosclerosis is a major risk factor of ischemic stroke [101]. Beyond depicting the lumen of smaller caliber intracranial arteries, research has leveraged the advantages of ultrahigh field imaging to evaluate directly the intracranial vessel wall [102–104]. MRI at 7 T was superior to 3-T MRI in vessel wall characterization and visualization of the fibrous cap and lipid core within atherosclerotic plaques in patients with intracranial atherosclerotic disease [105], suggesting the potential role of 7-T MR in risk stratification in intracranial atherosclerosis. Another study correlating *in vitro* intravascular sonography and 7-T MRI findings with histology concluded that 7-T MRI is a reliable method for detecting atherosclerotic burden within the intracranial arteries [106]. A recent study in 130 patients with a history of vascular diseases examined the association between intracranial atherosclerosis burden measured with intracranial vessel wall sequences at 7 T and several markers of extracranial atherosclerosis and found that ankle brachial index, the presence of extracranial carotid stenosis, carotid intima-media thickness, and decreasing glomerular filtration rate were all associated with a higher intracranial atherosclerotic burden, suggesting that similar atherogenic mechanisms underlie both processes [104]. Lindenholz et al. [102] investigated the association between intracranial vessel wall lesion burden and vascular risk factors in patients with ischemic stroke or transient ischemic attack of the anterior circulation. The authors reported that other than smoking, established common risk factors such as increasing age, diabetes, and hypertension were associated with a higher number and greater enhancement of intracranial vessel wall lesions in these patients. The same group used 7-T MRI to demonstrate differences in the appearance of the vessel wall in patients who underwent thrombosuction treatment after ischemic stroke compared to patients with stroke who did not undergo thrombectomy. The clinician should be aware of these differences to avoid misinterpretation on post-treatment follow-up imaging [103].

The increased SNR, higher spatial resolution, and better fluid suppression seen at 7 T have been leveraged to evaluate patients with aneurysms that are at high risk for rupture, predict potential for atherosclerotic plaque rupture, and characterize infarct morphology [52]. For example, Sato et al. used gadolinium-enhanced T1-weighted magnetization prepared rapid gradient recalled echo (MPRAGE) sequences at 7 T to describe aneurysm wall microstructures responsible for gadolinium enhancement not seen at lower field strengths. Partial or complete inner wall enhancement correlated with neovascularization of the inner wall layer as well as the adjacent thrombus, and partial or complete outer wall enhancement could be explained by formation of vasa vasorum in the outer aneurysm wall layer [107]. The authors concluded that the double-rim enhancement seen at 7 T correlated with perifocal edema and wall histologic findings suggestive of instability. In another study that compared 7 T and 1.5 T, the detection and characterization of unruptured aneurysms was demonstrated to be better at 7 T [108]. Wrede et al. [109] demonstrated that 7-T TOF MR images were comparable to digital subtraction angiography, the current gold standard.

In summary, ultrahigh field vessel wall imaging in the evaluation of cerebrovascular disease may enable earlier diagnosis, better differentiation of the underlying disease processes, and provide valuable information in the management intracranial vascular disease. However, the exclusion of DWI limits use of 7-T MRI in the evaluation of acute stroke patients at this time.

## Ultra-high and ultrafast gradient technology

### Technological overview and state-of-the-art

As a major component of the scanner, the gradient system plays a key role in MR image generation by enabling spatial selection and image encoding. The performance characteristics of the gradient system are parameterized by the maximum gradient amplitude, which is measured in mT/m, and the slew rate, which describes how fast a gradient can attain a desired amplitude within a given amount of time (T/m/s). The magnetic field gradients have tremendous influence on the overall quality of the acquired image, a point that has gained increasing focus from the major scanner manufacturers in the quest for improved image quality and ever-faster image encoding speed. Considerable engineering efforts have been made in the last 30 years to maximize the performance of gradient systems for clinical use. The most recent generation of commercial scanners such as the Siemens Prisma, GE Premier, and Philips Achieva 3T X-series (Quasar Dual gradient system) feature integrated whole-body gradient systems with a maximum gradient amplitude of 80 mT/m and maximum slew rates of 200, 200, and 100 T/m/s, respectively.

For applications such as diffusion MRI, gradient coils capable of producing gradient fields with high amplitudes significantly reduce the time required for diffusion encoding and improve the SNR of such measurements by reducing signal decay from T2 relaxation. Diffusion MRI has been the driving motivation behind recent efforts to engineer dedicated gradient coils with large maximum gradient amplitudes. For example, customized gradient coil inserts such as the Siemens SC72 gradient insert that was designed for the Washington University-Minnesota Consortium Human Connectome Project scanner are capable of generating maximum gradient strengths of 100 mT/m [110]. The Connectom whole-body gradient coil designed for the Massachusetts General Hospital-University of Southern California Human Connectome Project utilized a segmented design with 12 total gradient amplifiers (four 900 A drivers at 2250 V each per physical axis) to overcome efficiency limits and attain a maximum gradient amplitude of 300 mT/m and maximum slew rate of 200 T/m/s within a 56-cm bore diameter [111]. Smaller diameter head gradient coils are a viable alternative to achieving even higher maximum gradient amplitudes compared to whole-body gradient coils and have remained a design of interest for integration into 3-T MRI systems using clinically available gradient power amplifiers. As an example, the MAGNUS (Microstructure Anatomy Gradient for Neuroimaging with Ultrafast Scanning) head gradient coil offers 200 mT/m maximum gradient amplitude and slew rate of up to 500 T/m/s per axis within a 37-cm bore diameter using lower power amplifiers (620 A/1,500 V) than those used in the Connectom scanner [112].

### Challenges and opportunities of imaging with higher performance gradient systems

Higher performance gradient systems equipped with higher maximum gradient amplitudes and slew rates enable increased spatial resolution and faster acquisitions. To achieve higher spatial resolution, larger gradient areas are required, noting that the maximum area under a gradient encoding pulse determines the highest achievable spatial resolution. This can be achieved using lower gradient strengths by lengthening the gradient encoding pulses without increasing their amplitudes; unfortunately, this approach leads to a loss of SNR due to T2-related signal decay during the image readout period. To shorten the image readout time without sacrificing resolution, the gradient amplitude must increase, and the time to achieve the desired gradient amplitude must be minimized. High-performance gradient systems are thus designed to maximize gradient amplitude and slew rate within technical and biological limits. Shortening the gradient pulses during image readout must be carefully balanced against decreasing the SNR of the image beyond an acceptable limit, as the SNR is inversely related to the square root of the bandwidth, which is set by the gradient amplitude.

Technical limitations include the engineering challenges of achieving a spatially linear magnetic gradient field, removal of heat from the gradient coil, and efficiency and power requirements of the coil. While these technical challenges have largely been addressed in the modern era, including advances in gradient and shim technology [113–115], the main limiting factor on gradient coil performance is the biological effect of rapidly switching, large magnetic fields on the human body. Peripheral nerve stimulation (PNS) can be induced if the product of the gradient switching rate and strength generate electric fields large enough to trigger nerve firing, which can be uncomfortable and potentially painful. Due to such safety concerns, peripheral nerve stimulation is strictly regulated in MRI. The incorporation of anatomical models of the peripheral nerves into electromagnetic and neurodynamic simulations of PNS [116] promises to enable more accurate prediction of PNS thresholds and may inform the design of gradient coils to minimize PNS [117]. Smaller head gradients covering only the head and neck regions eliminate the induction of current paths in the chest and back, which are particularly prone to stimulation, and offer another strategy for reducing PNS in brain imaging [118].

### Clinical applications of ultrahigh gradient technology

#### Anatomic MRI

Stronger and faster gradients benefit anatomical imaging by enabling more efficient image readout, with a concomitant reduction in echo time and repetition time, depending on the image contrast of interest. The shortened acquisition time can then be used to increase spatial resolution and/or SNR through signal averaging within a similar overall scan time. For example, using the Connectome whole-body gradient system with a maximum gradient strength of 300 mT/m and slew rate of 200 T/m/s, and comparing against a whole-body gradient system with 60 mT/m and 200 T/m/s maximum gradient strength and slew rate, improvements in anatomical imaging include ~ 25% reduction in the repetition time and ~ 50% reduction in echo time for the 3D T1-weighted MPRAGE sequence due to shorter echo spacing, which translates into a roughly ~ 25% reduction in acquisition time.

**Fig. 7:**
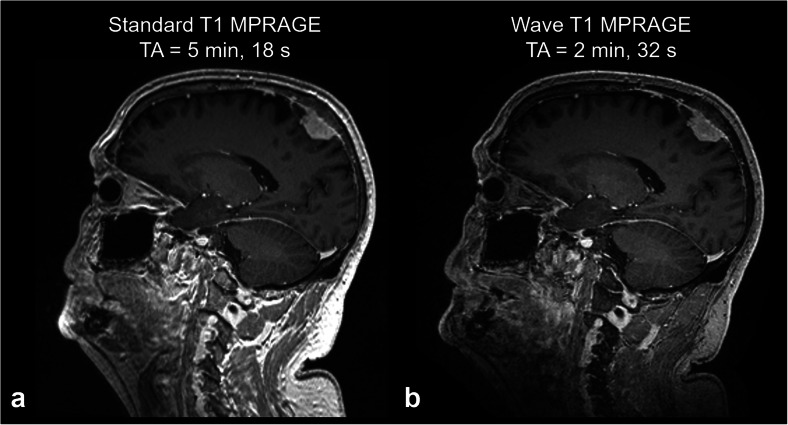
Comparison of post-contrast three-dimensional T1-weighted magnetization prepared rapid acquisition gradient recalled echo (MPRAGE) images acquired with conventional parallel imaging and wave-controlled aliasing in parallel imaging (CAIPI) encoding demonstrating a meningioma. Standard (**a**) and Wave-CAIPI (**b**) T1-weighted MPRAGE images show equivalent visualization of the dural-based enhancing mass along the parietal convexity. Both images were acquired on a 3-T Siemens Prisma MRI scanner equipped with 80 mT/m maximum gradient strength and 200 T/m/s maximum slew rate. The Wave-CAIPI sequence was more than twice as fast as the standard sequence (acquisition time = 2 min, 32 s for Wave-CAIPI compared to 5 min, 18 s for the standard sequence)

The constant push for shorter scan times has led to the development of more efficient k-space sampling schemes for 2D and 3D imaging, which benefit from better gradient performance. Spiral k-space encoding makes efficient use of the gradient system hardware [119] and has been used to achieve ultrashort echo times for real-time and rapid imaging applications [120]. As another example, wave-controlled aliasing in parallel imaging (CAIPI) is a 3D parallel imaging technique that uses a controlled pattern of gradient modulation in the phase- and partition-encoding directions to create 2D CAIPI shifts between partitions and voxel spreading in all three dimensions using sinusoidal gradients [121, 122]. Wave-CAIPI takes full advantage of the 3D coil sensitivity information when using high-channel count array coils to provide high acceleration factors with negligible artifacts and g-factor penalty across a variety of contrasts. In general, higher gradient amplitudes and lower bandwidth generate more voxel spreading [123], and higher slew rates allow for an increased number of sinusoidal cycles per encoding period, which diminish the amount of artifact in the resulting images. Wave-CAIPI takes advantage of higher gradient strengths and fast slew rates to enable higher resolution and faster anatomical imaging. The diagnostic performance of Wave-CAIPI has been shown to be equivalent to that of standard 3D anatomical sequences acquired with conventional parallel imaging across a variety of contrasts with reduced scan time and motion artifacts [123–126] (Fig. 7).

#### Diffusion MRI

High-performance gradient systems equipped with large maximum gradient amplitudes are particularly beneficial for diffusion MRI as they boost the efficiency of diffusion encoding. The use of stronger gradients enables a given diffusion-encoding gradient area to be achieved in less time and effectively shortens the entire diffusion-encoding period and echo time, resulting in less signal loss from T2 relaxation. Beyond the increase in SNR, larger gradient amplitudes enable stronger diffusion encoding to be achieved with shorter effective diffusion times, which improves the sensitivity of the diffusion MRI measurement to microscopic structures on the micron scale [127] and improves the resolution of fine structures such as crossing fibers for diffusion tractography [128, 129]. The availability of dedicated high-performance gradient coils with large maximum gradient amplitudes enables new classes of diffusion MRI measurements to be performed in the living human brain and provides a more sensitive probe of white matter microstructure in various neurological diseases. High gradient amplitudes benefit the estimation of tissue microstructural properties such as neurite density and orientation dispersion [130], axon diameter [131–135], and the dimensions of the extracellular space [136], which offer greater specificity to tissue microstructure changes than diffusion tensor imaging.

Among the tissue microstructural properties that can be measured using diffusion MRI, axon diameter estimation requires high gradient amplitudes to sensitize the diffusion MRI signal to intra-axonal water diffusion [134, 137–139]. Axon diameter mapping using high-gradient diffusion MRI has been applied to study changes in axonal structure and packing density in MS and the aging brain. Axon diameter mapping in the corpus callosum of relapsing-remitting and progressive MS patients has revealed increased axon diameter and decreased axon density in lesions and the normal-appearing white matter [140–142], in keeping with findings on histopathology, which show decreased axon density and an overall increase in axon diameter in postmortem MS tissue [143]. The observed increase in axon diameter as measured by high-gradient diffusion MRI is also a strong predictor of disability and cognitive dysfunction [144], particularly in tests of interhemispheric processing speed and working memory, which rely on the corpus callosum as the major white matter fiber tract mediating these processes. The application of axon diameter mapping to the aging brain has uncovered significant increases in axon diameter with advancing age in the anterior white matter [145], with changes most pronounced in the genu of the corpus callosum and forceps minor. These alterations in axon diameter are consistent with previously reported regional decreases in fractional anisotropy within the frontal white matter [146] and parallel decreases in corpus callosum area and regional gray matter volume with age.

The availability of ultrastrong gradients for diffusion MRI has enabled the translation of novel diffusion-encoding paradigms to probe brain tissue microstructure in patients, including oscillating-gradient waveforms [147], double-diffusion encoding [148], and q-space trajectory imaging [149]. One recent study combined isotropic diffusion encoding with ultrastrong diffusion gradients to achieve high diffusion-weighting in highly restricted, spherical compartments (the so-called dot compartment) in the cerebellar gray matter while suppressing signal arising from anisotropic water within axons [150]. By gaining greater specificity to cellular signatures in the cerebellum, spherical tensor encoding performed with high gradient strengths may enable the earlier identification of cerebellar gray matter loss in patients with hereditary ataxias such as spinocerebellar ataxia type 2, which selectively affects the granule and Purkinje cells. The ability to probe microscopic diffusion anisotropy in brain tumors using q-space trajectory imaging may enable the differentiation of brain tumors such as meningiomas from high-grade glial tumors based on their cellular morphology and composition [151].

The results of clinical research studies demonstrate the potential of high-gradient diffusion MRI to uncover changes in axonal and cellular microstructure and motivate the continued development, application, and dissemination of high-gradient technology for use in commercially available human MRI scanners.

#### Functional MRI

Higher gradient strengths and faster slew rates facilitate the development and application of accelerated fMRI acquisitions at high spatial resolution. Higher gradient strengths and faster slew rates translate directly into better image quality for echo-planar imaging, the workhorse of functional MR image readout, by boosting the SNR, reducing distortions, and enabling higher in-plane resolution and thinner slices. Beyond the benefits to image quality, the fast slew rates available on high-performance gradient systems promise to enhance the acquisition efficiency and dense sampling of T2 and T2* signal evolution for fast quantitative mapping of the BOLD signal arising from the micro- and microvasculature. Acquiring fMRI data at higher temporal and/or spatial resolution promises to provide new information about neuronal activation, particularly in the early phases of the BOLD response that are thought to reflect the fast response of smaller blood vessels in the brain to local neuronal activity. Traditionally, fMRI has been thought to be too slow to measure brain oscillations due to the dependence of the fMRI signal on slow changes in blood flow. However, recent studies using ultrafast fMRI at 3 and 7 T have revealed fast neural oscillations at up to 0.75 Hz in the visual cortex following high-frequency visual stimulation [152, 153]. Although it remains to be seen whether these signals carry additional information beyond that embedded in the slower BOLD oscillations, the possibility of acquiring such signals promises to uncover abnormal fluctuations in small deep brain structures, such as brainstem nuclei, while correcting for rapid physiological noise (*e.g.*, cardiac and respiratory motion around the brainstem). Given that the pathophysiological hallmark of Parkinson’s disease is a degeneration of several neurotransmitter systems in the brain stem, these new techniques may make it possible to test for altered activity and functional connectivity of these nuclei in PD and other neurodegenerative disorders.

## Conclusions

MRI technology development over the last 20 years has focused on pushing the limits of static magnetic field strength and gradient amplitude and speed. Advances in imaging at ultrahigh field, particularly at 7 T, have effectively ushered in next-generation image quality for clinical neuroimaging, with better SNR and higher resolution scans that provide better visualization of small and subtle lesions such as cortical lesions in MS and cortical malformations in epilepsy. At the same time, advances in gradient technology have enabled high-fidelity image acquisition within shorter scan times, and have also enabled better resolution of tissue microstructure through diffusion MRI, allowing for the first time the characterization of microscopic tissue features in the living human brain paralleling what can be seen directly on histopathology. Building on the success of these advances, the next few decades will likely see a continued push toward even higher field strengths and higher gradient strength systems, while encouraging the more widespread adoption of ultrahigh field and ultrahigh, ultrafast gradient technology for clinical use.

## Data Availability

Not applicable.

## Abbreviations

3D: Three-dimensional
AD: Alzheimer’s disease
APT: Amide proton transfer
BOLD: Blood oxygenation level dependent
CA: Cortical area
CAIPI: Controlled Aliasing in Parallel Imaging
CEST: Chemical exchange saturation transfer imaging
CVS: Central vein sign
FLAIR: Fluid attenuation inversion-recovery
fMRI: Functional magnetic resonance imaging
IDH: Isocitrate dehydrogenase
MAP: Morphometric analysis program
MPRAGE: Magnetization prepared rapid gradient recalled echo
MR: Magnetic resonance
MRI: Magnetic resonance imaging
MS: Multiple sclerosis
NOE: Nuclear Overhauser effect
PD: Parkinson’s disease
PNS: Peripheral nerve stimulation
rNOE: Relayed NOE
SAR: Specific absorption rate
SNR: Signal-to-noise ratio
SWI: Susceptibility-weighted imaging
TOF: Time of flight
